# Supplementary material for: Determinants of anthroponotic cutaneous leishmaniasis by case-control study in Morocco
Source: PLoS One. 2022 Oct 14;17(10):e0266291. doi: 10.1371/journal.pone.0266291 (PMC9565455; doi:10.1371/journal.pone.0266291)
Supplement: Attachment — Submitted filename: Response Letter PLOS.docx [file pone.0266291.s002.docx]

**RESPONSE LETTER**

PONE-D-22-07886

Determinants of Anthroponotic Cutaneous Leishmaniasis by Case-Control Study in Morocco

PLOS ONE

Dear Editor,

Thank you for the opportunity to submit a revised version of our manuscript entitled “**Determinants of Anthroponotic Cutaneous Leishmaniasis by Case-Control Study in Morocco**”. We appreciate the time and efforts you and the reviewers have put into providing your valuable comments on our manuscript. We are grateful for your insightful comments on our paper. We were able to incorporate changes to reflect all the suggestions provided.

We have highlighted the changes in the manuscript.

Sincerely,

Pr S. BOUSSAA

Corresponding author

**Journal Requirements:**

When submitting your revision, we need you to address these additional requirements.

1. Please ensure that your manuscript meets PLOS ONE's style requirements, including those for file naming. The PLOS ONE style templates can be found at 
<https://journals.plos.org/plosone/s/file?id=wjVg/PLOSOne_formatting_sample_main_body.pdf> and 
<https://journals.plos.org/plosone/s/file?id=ba62/PLOSOne_formatting_sample_title_authors_affiliations.pdf>

**Authors:** The revised manuscript was formatted according to PLOS ONE's style

2. Please provide additional details regarding participant consent. In the ethics statement in the Methods and online submission information, please ensure that you have specified what type you obtained (for instance, written or verbal, and if verbal, how it was documented and witnessed). If your study included minors, state whether you obtained consent from parents or guardians. If the need for consent was waived by the ethics committee, please include this information.

**Authors:** The ethics statement section has been rewritten, based on comments from the editor and reviewer, to be more informative.

3. Please amend either the abstract on the online submission form (via Edit Submission) or the abstract in the manuscript so that they are identical.

**Authors:** The abstract on the online submission was updated.

4. We note that Figure 1 in your submission contain [map/satellite] images which may be copyrighted. All PLOS content is published under the Creative Commons Attribution License (CC BY 4.0), which means that the manuscript, images, and Supporting Information files will be freely available online, and any third party is permitted to access, download, copy, distribute, and use these materials in any way, even commercially, with proper attribution. For these reasons, we cannot publish previously copyrighted maps or satellite images created using proprietary data, such as Google software (Google Maps, Street View, and Earth). For more information, see our copyright guidelines: <http://journals.plos.org/plosone/s/licenses-and-copyright>.
 We require you to either (1) present written permission from the copyright holder to publish these figures specifically under the CC BY 4.0 license, or (2) remove the figures from your submission:
 a) You may seek permission from the original copyright holder of Figure 1 to publish the content specifically under the CC BY 4.0 license.  
 We recommend that you contact the original copyright holder with the Content Permission Form (<http://journals.plos.org/plosone/s/file?id=7c09/content-permission-form.pdf>) and the following text:
“I request permission for the open-access journal PLOS ONE to publish XXX under the Creative Commons Attribution License (CCAL) CC BY 4.0 (<http://creativecommons.org/licenses/by/4.0/>). Please be aware that this license allows unrestricted use and distribution, even commercially, by third parties. Please reply and provide explicit written permission to publish XXX under a CC BY license and complete the attached form.”
 Please upload the completed Content Permission Form or other proof of granted permissions as an "Other" file with your submission.
In the figure caption of the copyrighted figure, please include the following text: “Reprinted from [ref] under a CC BY license, with permission from [name of publisher], original copyright [original copyright year].”
 b) If you are unable to obtain permission from the original copyright holder to publish these figures under the CC BY 4.0 license or if the copyright holder’s requirements are incompatible with the CC BY 4.0 license, please either i) remove the figure or ii) supply a replacement figure that complies with the CC BY 4.0 license. Please check copyright information on all replacement figures and update the figure caption with source information. If applicable, please specify in the figure caption text when a figure is similar but not identical to the original image and is therefore for illustrative purposes only.
The following resources for replacing copyrighted map figures may be helpful:
USGS National Map Viewer (public domain): <http://viewer.nationalmap.gov/viewer/>
The Gateway to Astronaut Photography of Earth (public domain): <http://eol.jsc.nasa.gov/sseop/clickmap/>
Maps at the CIA (public domain): <https://www.cia.gov/library/publications/the-world-factbook/index.html> and <https://www.cia.gov/library/publications/cia-maps-publications/index.html>
NASA Earth Observatory (public domain): <http://earthobservatory.nasa.gov/>
Landsat: <http://landsat.visibleearth.nasa.gov/>
USGS EROS (Earth Resources Observatory and Science (EROS) Center) (public domain): [http://eros.usgs.gov/#](http://eros.usgs.gov/)
Natural Earth (public domain): <http://www.naturalearthdata.com/>

**Authors:** Figures 1 and 2 were performed by authors; consequently we can publish it on open access form under CC BY 4.0. We used free sheapfile from <https://mapcruzin.com/free-morocco-arcgis-maps-shapefiles.htm>, for map creation (Fig. 1).

Figures were presented under TIF format and titles were modified according to the journal recommendations’

**Reviewer #1:**

In the current study, some important determinants of local anthroponotic cutaneous leishmaniasis are investigated in Morocco.

Introduction:
Line 47: At the beginning of each sentence the scientific names must be in complete form (L. tropica must be changed to Leishmania tropica).

**Authors:**

Thank you to the Reviewer for the positive feedback and useful comments. This remark has been corrected throughout the manuscript.

Results:
There are some errors in table 2 which must be corrected (in Gender line after parenthesis if cases”1” must be deleted. In the line of level of study, “Literary” is misspelled).

**Authors:** Tables were modified according to Reviewer comments

Sandfly/sandflies should be written separately (sand fly/ sand flies) in the manuscript.

Line 272: Scientific names must be written in italic form (P. sergenti must be in italic).
**Authors:** These remarks have been corrected throughout the manuscript.

Discussion:
Lines 212-223 are not related to the discussion. These sentences can be included in the introduction.
The results should not be repeated in discussion. All p values in the discussion should be omitted. In discussion, the results should be compared with the results of other studies and described.
In the last paragraph of the discussion (lines: 293-301) all factors should be included. Using mosquito nets is missed.
There are many valuable published articles by Iranian scientists. It is recommended that the authors of the current manuscript use them for the introduction and results. For example for the effect of mosquito nets on cutaneous leishmaniasis the following references can be used:
-Moosa-Kazemi SH, Yaghoobi-Ershadi MR, Akhavan AA, Abdoli H, Zahraei-Ramazani AR, Jafari R, Houshmand B, Nadim A, Hosseini M. (2007) Deltamethrin-impregnated bed nets and curtains in an anthroponotic cutaneous leishmaniasis control program in northeastern Iran. Ann Saudi Med. 27(1): 6–12.
- Yaghoobi-Ershadi MR, Moosa-Kazemi SH, Zahraei-Ramazani AR, Jalali-Zand AR, Akhavan AA, Arandian MH, Abdoli H, Houshmand B, Nadim A, Hosseini M. (2006) Evaluation of deltamethrin-impregnated bed nets and curtains for control of zoonotic cutaneous leishmaniasis in a hyperendemic area of Iran. Bull Soc Pathol Exot. 99(1): 43–48.

**Authors:** Thank you for these valuable remark and resources. The Discussion section was reviewed according to the comments and all references were included. Please see the revised version of the manuscript

**Reviewer #2:**

Line 15: "Are" should be replaced with "is".

Line 38: "Are" should be replaced with "is".
**Authors:** Thank you to the Reviewer for the positive feedback and useful comments. These remarks were fixed

Line 28: Whether absent of sewage system is beneficial or its presence? its unclear. Please rephrase the sentence.

**Authors:** According to our results, the absent of sewage system/waste management increases the risk of ACL which means that its presence is beneficial (protective factor). This sentence was reviewed in the revised version of the manuscript.

Line 54: Here the reference, Boussaa et al is not cited properly, i.e. by numbering. Please do the needful.

**Authors:** Thank you for this comment. The manuscript was revised according to the journal style.

Line 99-103: Please explain more about sample size calculation. It is not well explained at all.

**Authors:** We added a paragraph in material and methods section about sample size calculation in this case- control study.

Line 119-122: How the data was tabulated and how categorization was done? This part can be more explained for better understanding, like what is the process of categorization etc.

**Authors:** We added a paragraph at our opining paragraph of the material and methods section to help the reader to undesrstand : “The questionnaire has three sections covering socio-demographic (Gendre, Age, Matrimonial status, Level of study, knowledge about leishmaniasis), socioeconomic (Number of children per family, Person per household, Provinces, Monthly income (dhs), Zone, Travel to endemic area, Profession) and environmental data (Sewage system/waste management, Toilet/ Waste water management, Mosquito nets, Vegetation, Farm animals, Companion animals). The categorisation was based on previous studies exploring the factors of leishmaniasis in all its forms (28) (29)”. Please see the revised version of the manuscript.

Line 136: Please mention whether the consent taken is written or verbal.

**Authors:** The ethics statement section has been rewritten, based on comments from the editor and reviewer, to be more informative.

Line 147: Change the phrase as : "females are more affected".

Line 202: Rephrase the sentence as : presence of vegetation.

**Authors:** Phrases were changed accordantly

Line 193-195: Sentence is not properly understood. Please re-phrase the sentence.

**Authors:** This section was modified according to the Reviewer comments. Please see the revised version of the manuscript.

Line 224: Use of reference 27 is not understood. You have sated about your study findings here, so how reference 27 is appropriate here?

**Authors:** This paragraph was rephrased to be clearer.

Line 230: How is this associated? Negatively or positively?

**Authors: T**his is associated negatively, we rectified it in manuscript

Line 267-275: This part could be better explained, why there is such finding, whats the reason behind it.
**Authors:** This part was developed and more explained according to the reviewer comment.

Line 278-279: Please explain.

**Authors:** This paragraph was rephrased to be clearer. Please see the revised version of the manuscript.
